# Correlation between metabolic syndrome and periurethral prostatic fibrosis: results of a prospective study

**DOI:** 10.1186/s12894-024-01413-y

**Published:** 2024-02-12

**Authors:** Jingwen Ren, Yuanyuan Li, Xueyuan Zhang, Min Xiong, Heng Zhang, Lingyue An, Ying Cao, Shujie Xia, Guangheng Luo, Ye Tian

**Affiliations:** 1 Department of Urology, Guizhou Provincial People’s Hospital, Guiyang, China; 2 Department of Urology, The First Affiliated Hospital of Chongqing Medical University, Chongqing, China; 3 Guizhou University School of Medicine, Guiyang, China; 4 Zunyi Medical University, Zunyi, China; 5 Department of Urology, Shanghai General Hospital, Shanghai Jiaotong University, Shanghai, China

**Keywords:** Prostatic fibrosis, Metabolic syndrome, Lower urinary tract symptoms

## Abstract

**Background:**

Prostatic fibrosis, characterized by the accumulation of myofibroblasts and collagen deposition, is closely associated with LUTS and may lead to mechanical obstruction of the urethra. Additionally, Metabolic Syndrome (MetS), characterized by central obesity, high blood sugar, lipid metabolism disorders, and hypertension, is increasingly recognized as a proinflammatory condition linked to prostate inflammation.

**Methods:**

Clinical data from 108 subjects who underwent transurethral resection of the prostate or bipolar plasmakinetic enucleation of the prostate were prospectively collected between June 2021 and August 2022. Patients were divided in two groups according to whether or not they had a diagnosis of MetS. Specimens were stained with Masson trichrome and the periurethral prostatic fibrosis extent was evaluated using quantitative morphometry.

**Results:**

Forty-three patients (39.8%) were diagnosed with MetS. Patients with MetS showed a significantly greater extent of prostatic fibrosis than the others (68.1 ± 17.1% vs. 42.5 ± 18.2%, *P* < 0.001), and there was a positive correlation between the number of positive MetS parameters and the extent of prostatic fibrosis (*R*^2^ = 0.4436, *P* < 0.001). Multivariate regression analysis revealed that central obesity (B = 2.941, 95% confidence interval, 1.700–3.283), elevated fasting glucose (B = 1.036, 95% confidence interval, 0.293–1.780), reduced HDL cholesterol (B = 0.910, 95% confidence interval, 0.183–1.636) and elevated triglycerides (B = 1.666, 95% confidence interval, 0.824–2.508) were positively correlated to prostatic fibrosis. Elevated blood pressure, however, was unrelated to prostatic fibrosis (B = 0.009, 95% confidence interval, -0.664–0.683).

**Conclusions:**

The present findings suggest that prostatic fibrosis is positively correlated with MetS and its components including central obesity, elevated fasting glucose, reduced high density lipoprotein cholesterol and elevated triglycerides.

## Introduction

Lower urinary tract symptoms (LUTS) are highly prevalent worldwide, particularly after the age of 50 years [1, 2]. LUTS form a progressive disorder manifesting as urgency, nocturia, urinary frequency, a weak urinary stream and incomplete bladder emptying and are concurrently associated with sleep quality, depression, ill health and even mortality [1, 3–5]. Benign prostatic hyperplasia (BPH) is regarded as the leading cause of LUTS in elderly males due to bladder outlet obstruction (BOO) attributable to an enlarged prostate. However, in clinical practice, approximately half of BOO patients have a relatively small prostate (30 mL or less) [6]. Moreover, in a considerable number of patients, the progression of LUTS is refractory to 5α-reductase inhibitors and/or α1-adrenergic receptor antagonists [5] but often responds well to simple prostatectomy, indicating the heterogeneity of this disease.

Fibrosis is characterized by myofibroblast accumulation, collagen deposition, and tissue stiffening. Ma et al. [7] showed that periurethral prostatic fibrosis was significantly associated with tissue stiffness as well as LUTS. Such pathological changes could decrease urethral flexibility and trigger mechanical obstruction during micturition. Tissue inflammation is an important trigger of organ fibrosis. Cantiello and colleagues indicated a positive association between LUTS and collagen content introduced by inflammation [8]. Although the role of prostatic fibrosis in LUTS is recognized, its etiology remains poorly understood.

Metabolic syndrome (MetS) is defined by components including central obesity, high blood glucose, disordered lipid metabolism and hypertension. Over the past two decades, the prevalence of MetS has increased worldwide, especially in the elderly population [9]. MetS has been considered a proinflammatory disease [10, 11] and MetS may change the environment of microbiota in the prostate, and the change of microbiota may be the biological mechanism of prostatitis [12]. Growing evidence suggests that MetS represents an independent risk factor for prostate inflammation [13–15], which might eventually contribute to periurethral prostatic fibrosis and LUTS [16].

The aim of this clinical study was to investigate the correlation of periurethral prostatic fibrosis and MetS, as well as to identify the responsible MetS components using a prospective cohort.

## Patients and methods

### Patients

The study protocol of the current analysis was reviewed by the institutional ethics committee (No. 2021–67), and informed consent was obtained from each patient or the legal guardian. The data of a consecutive series of BPH patients treated with transurethral resection of prostate (TURP) or bipolar plasmakinetic enucleation of the prostate (PKEP) between June 2021 and August 2022 were prospectively collected at the Guizhou Provincial People’s Hospital and Shanghai General Hospital, Shanghai Jiaotong University.

The subjective symptoms of all patients were scored according to the international prostate symptom score (IPSS) and quality of life (QoL) score. In addition, physical examinations, including digital rectal examination (DRE), prostate-specific antigen (PSA) level, transabdominal kidney-bladder ultrasound, transrectal ultrasound (TRUS) (Philips EPIQ 5 ultrasound machine, Amsterdam, Netherlands), post-void residual urine (PVR), and maximum flow rate (Q_max_), were performed for all patients. Total prostate volume (TPV) was calculated from TRUS measurements of the prostate using the prostate ellipsoid formula (height × width × length × π/6). Patients with suspected prostate cancer also underwent ultrasound-guided transrectal 12-core needle biopsy to confirm the BPH diagnosis.

The exclusion criteria were as follows: incomplete medical records; previous bladder, prostate, or urethra operation; urethral stricture; IPSS ≤ 7; prostate or bladder cancer; bladder stones; acute urinary tract infection; neurogenic bladder dysfunction and any comorbidities that could affect the patient’s voiding function.

### Definition of MetS

MetS was defined by at least three of the following five components: elevated blood pressure (BP ≥ 130/85 mmHg), central obesity (WC > 102 cm), elevated triglycerides (≥ 1.7 mmol/L), elevated fasting glucose (≥ 5.6 mmol/L) and reduced high density lipoprotein (HDL) cholesterol (< 1.0 mmol/L) [17–19]. Waist circumference (WC) and BP were measured by trained personnel using a standardized protocol. In particular, WC was measured midway between the lowest rib and the iliac crest to the nearest 1 cm. Blood samples were drawn in the morning preoperatively, after an overnight fast, for measurement of blood glucose, HDL cholesterol and triglycerides.

### Tissue procurement and preparation

**Fig. 1:**
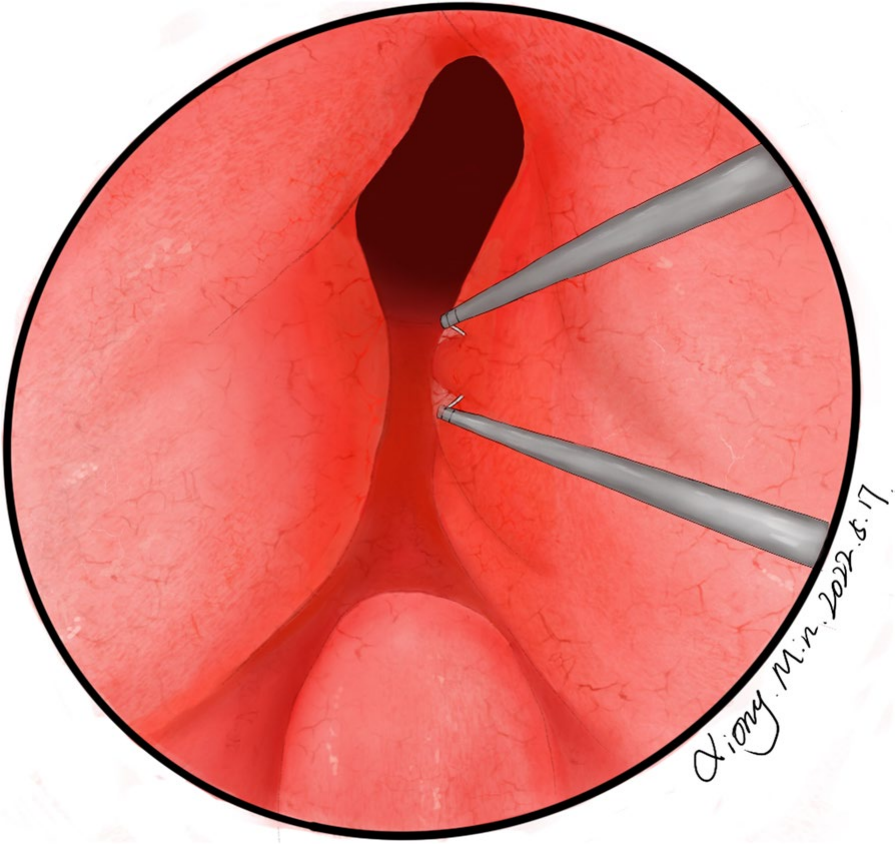
Schematic diagram of tissue procurement. The tissue procurement started at the 3 o’clock position on the left lobe of the prostate, prior to the TURP/PKEP procedure, approximately in the middle of the bladder neck and the verumontanum

TURP or PKEP was performed using the ScanMed Plasmakinetic System (ScanMed, Zhuhai, China) with plasma electrodes set at 160 W for cutting and 80 W for coagulating. Periurethral prostate specimens were obtained prior to the procedures. Briefly, the tissue procurement started at the 3 o’clock position on the left lobe of the prostate, approximately in the middle of the bladder neck and the verumontanum, which guaranteed urothelium in the harvested tissue (Fig. 1). Then, the periurethral prostate specimens were fixed in 10% paraformaldehyde and embedded in paraffin.

### Assessment of prostatic fibrosis

Two dedicated uropathologists, blinded to the clinical and laboratory findings, performed the pathological examinations. Specimens were removed from paraffin and stained with Masson’s trichrome as we previously described [20]. Prostatic fibrosis was assessed using quantitative morphometry (Olympus Software cell-Sens 1.6, Olympus, Tokyo, Japan) and defined as the blue area in Masson’s trichrome-stained tissue sections (section with positive staining for collagen) in relation to the total tissue area.

### Statistical analysis

Patients were divided into a MetS group and a non-MetS group. Data are expressed as the mean ± standard deviation (SD) and were compared between the two groups using an unpaired, 2-tailed t test. Pearson’s correlation analysis was used to test the association between the number of positive MetS parameters and prostatic fibrosis severity. All MetS components were included in multivariate logistic analysis to further evaluate their role in prostatic fibrosis. Statistical analysis was performed with GraphPad Prism version 8.0 (San Diego, CA, US) and SPSS 22.0 for Windows (Chicago, IL, US). A *P* value < 0.05 was considered statistically significant.

## Results

### Patient characteristics

Table 1 lists patients’ descriptive statistics stratified according to MetS diagnostic criteria. A total of 108 eligible patients were included for the analysis, of whom 43 patients (39.8%) were diagnosed with MetS. There were no significant differences between the two groups regarding age (*P* = 0.954), PSA level (*P* = 0.591), Q_max_ (*P* = 0.216), PVR (*P* = 0.299) or TPV (*P* = 0.358). Patients with MetS seemed to have a marginally higher IPSS (*P* = 0.104) and a favorable QoL score (*P* = 0.055); however, the differences were not significant.

**Table 1:** Patient characteristics and descriptive statistics, stratified according to their MetS profile

Variable	Overall	MetS (*n* = 108)		*P* value
		With (*n* = 43)	Without (*n* = 65)	
Age (y)	71.3 ± 7.6	71.2 ± 6.8	71.3 ± 8.7	0.954
PSA (ng/mL)	5.1 ± 4.8	4.8 ± 6.1	5.3 ± 3.8	0.591
IPSS	23.5 ± 6.4	24.7 ± 6.8	22.7 ± 6.1	0.104
QoL	4.6 ± 1.2	4.4 ± 1.3	4.8 ± 1.0	0.055
Q_max_ (mL/s)	6.4 ± 3.5	6.9 ± 3.7	6.0 ± 3.4	0.216
PVR (mL)	91.0 ± 128.0	106.8 ± 152.3	80.8 ± 108.9	0.299
TPV (mL)	56.8 ± 28.4	53.7 ± 29.3	58.8 ± 27.8	0.358

Values are presented as mean ± standard deviation

*MetS* Metabolic syndrome, *PSA* Prostate-specific antigen, *IPSS* International prostate symptom score, *QoL* Quality of life, *PVR* Postvoid residual, *TPV* Total prostate volume

### Periurethral prostatic fibrosis positively correlated with MetS

**Fig. 2:**
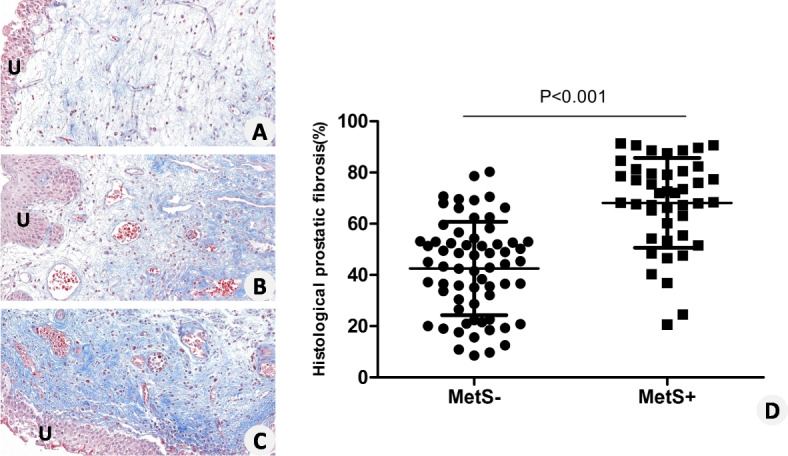
Periurethral prostatic fibrosis was determined by Masson’s trichrome staining. **A** Mild prostatic fibrosis. **B** Moderate prostatic fibrosis. **C** Severe prostatic fibrosis. **D** The prostate in the MetS group showed a significantly greater extent of fibrosis than that in the non-MetS group (68.1 ± 17.1% vs. 42.5 ± 18.2%, *P* < 0.001). U: urothelium

**Fig. 3:**
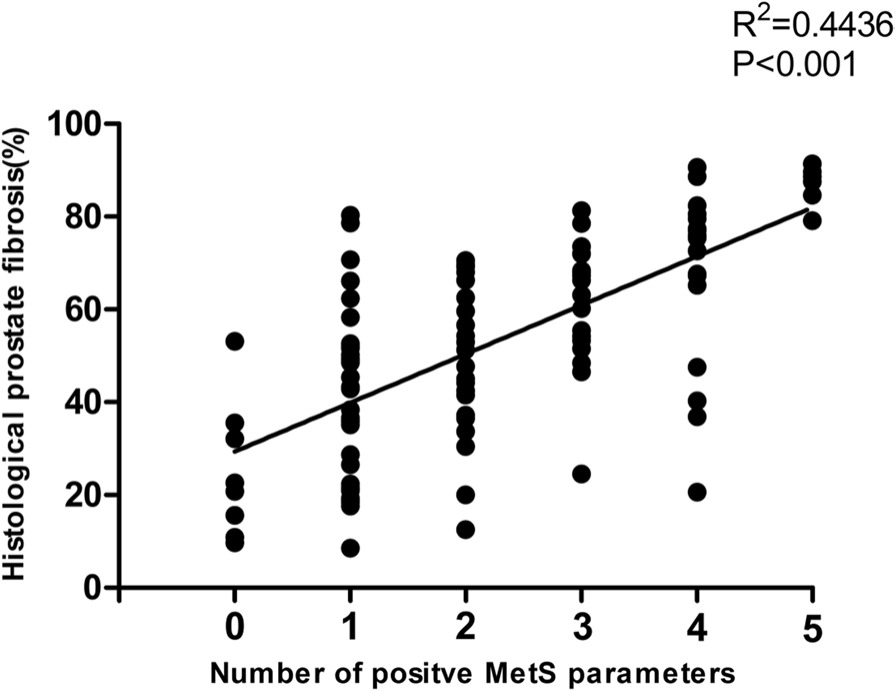
Correlation between prostatic fibrosis and the number of positive MetS parameters. There was a positive correlation between the number of positive MetS parameters and the extent of prostatic fibrosis (*R*^2^ = 0.4436, *P* < 0.001)

At the histological assessment, patients in the MetS group showed a significantly greater extent of prostatic fibrosis compared with those in the non-MetS group (68.1 ± 17.1% vs. 42.5 ± 18.2%, *P* < 0.001), which is shown in Fig. 2. To further study the relationship between MetS and prostatic fibrosis, a correlation analysis was performed. As shown in Fig. 3, Pearson’s correlation analysis demonstrated a positive correlation between the number of positive MetS parameters and the extent of prostatic fibrosis (*R*^2^ = 0.4436, *P* < 0.001). These results indicated that periurethral prostatic fibrosis positively correlated with both MetS and the number of positive MetS parameters.

### MetS components and periurethral prostatic fibrosis

All 5 MetS components were entered for multivariate regression analysis. As shown in Table 2, the analysis revealed that central obesity (B = 2.941, 95% confidence interval (CI), 1.700–3.283), elevated fasting glucose (B = 1.036, 95% CI, 0.293–1.780), reduced HDL cholesterol (B = 0.910, 95% CI, 0.183–1.636) and elevated triglycerides (B = 1.666, 95% CI, 0.824–2.508) were positively correlated with prostatic fibrosis. Elevated blood pressure, however, was unrelated to prostatic fibrosis (B = 0.009, 95% CI, -0.664–0.683).

**Table 2:** Multivariate regression analysis to evaluate the impact of each MetS component on prostatic fibrosis

MetS components	B (non-standardized coefficient)	Lower limit	Upper limit	*P* value
Central obesity	2.491	1.700	3.283	< 0.001
Elevated blood pressure	0.009	-0.664	0.683	0.978
Elevated fasting glucose	1.036	0.293	1.780	0.006
Reduced HDL cholesterol	0.910	0.183	1.636	0.014
Elevated triglycerides	1.666	0.824	2.508	< 0.001

*MetS* Metabolic syndrome

## Discussion

Both LUTS and MetS have become increasingly prevalent among elderly individuals, and a growing body of literature has suggested a possible correlation between LUTS and MetS. By 2018, an estimated 2.3 billion individuals will be affected by at least one LUTS (18.4% increase) [5]. Simultaneously, the United States witnessed a 35% increase in MetS prevalence from the appearance of the term in the 1980s to 2012 [21]. In the current study, 39.8% of the patients were diagnosed with MetS at an average age of 71.3 years. We found that periurethral prostatic fibrosis positively correlated with MetS and the number of positive MetS parameters. Moreover, multivariate regression analysis demonstrated that central obesity, elevated fasting glucose, reduced HDL cholesterol and elevated triglycerides were independently correlated with prostatic fibrosis.

The various pathogenic pathways contributing to the development of MetS culminate in a proinflammatory state, which is the connection between MetS and various diseases. Gacci et al. [22] reported that dyslipidemia might be considered the strongest proinflammatory factor among all MetS components, leading to proatherogenic remodeling of the vascular wall and ultimately resulting in tissue inflammation and postischemic tissue changes. Similarly, enlarged adipose cells from visceral adipose tissue in obese MetS patients produce increased concentrations of proinflammatory cytokines and chemokines, which are inextricably and biologically linked with inflammation and fibrotic changes [23]. Adipocyte cell size has been shown to be an independent predictor of insulin resistance and risk for type 2 diabetes, which was associated with the expression and secretion of important inflammatory molecules (such as TNFα and IL-6), further supporting the proinflammatory state [23]. Both human and mouse studies indicated inflammation as a common biological link among prostatic fibrosis, LUTS and MetS [13, 24–26]. It has been shown that adipokines interact with stromal and epithelial cells within the prostate, promoting a transdifferentiation of prostatic fibroblasts into myofibroblasts, along with abnormal cell growth. Through changes in the subclinical inflammatory state, MetS eventually leads to inflammation–stromal reorganization–fibrosis and prostatic hyperplasia [25].

Another possible mechanism involved in the correlation between prostatic fibrosis and MetS is sex hormone changes. We investigated the effects of estrogen and androgen on prostatic fibrosis using a rat model. Our previous results showed that an imbalance in the estrogen/androgen ratio may affect prostatic fibrosis and that estradiol may induce and aggravate the degree of prostatic fibrosis [20]. Enatsu et al. [27] found that rats that received oral administration of 0.1 mg/kg/day dutasteride showed fibrotic changes in the prostate but not changes in the hormonal or morphological status of the bladder. The changes were associated with altered expression of androgen and estrogen receptors. Studies have also documented histological changes in prostatic tissue architecture, including increased levels of inflammatory infiltrate and fibrosis, after androgen deprivation therapy [28]. Ali and colleagues [29] revealed that the mean testosterone level was significantly lower among patients with MetS, while the mean estradiol level was significantly higher among patients with MetS. The mean testosterone-to-estradiol ratio was significantly smaller among patients with MetS. Similarly, a correlation between sex hormone changes and BPH was also found in rabbits on a high-fat diet (HFD), and receptors for sex steroids were all increased in the prostates of rabbits fed a HFD [25].

Whether prostate volume is correlated with symptom scores and QoL scores remains unclear [30]. The Medical Therapy of Prostatic Symptoms study showed that among patients with small prostate volumes, only those with inflammation suffered from LUTS and experienced urinary retention episodes [31]. Whether MetS is associated with an increased prostate volume is controversial. Xia et al. [25] found that MetS and its pathological factors were associated with an increased PV and an increased risk of BPH/LUTS, and as the number of abnormal MetS components increased, the prostate volume presented a trend of progressive increase. The current study showed that there was no difference in prostate volume between patients with MetS and those without MetS. However, our cohort was highly selective because the included patients were surgery candidates. Cantiello et al. [13] indicated that MetS was not associated with an increased prostate volume. They speculated that MetS patients could experience LUTS secondary to chronic prostate inflammation and inflammation-dependent periurethral fibrotic changes, also regardless of the prostate volume, which we believe could be partially interpreted to mean that some patients suffered severe LUTS but were diagnosed with a small prostate.

Gharaee-Kermani et al. [24] sought to test whether prostatic fibrosis and LUTS would be exacerbated by diet-induced obesity and concurrent type 2 diabetes mellitus (T2DM) in a mouse model. They suggested that obesity, T2DM, lower urinary tract fibrosis, and urinary voiding dysfunction are inextricably and biologically linked. Cantiello and colleagues [13] investigated the pathological relationship between MetS and periurethral fibrosis status, and periurethral prostate tissue was obtained via biopsies carried out on radical prostatectomy specimens. They suggested that MetS might represent an independent risk factor for fibrotic changes secondary to inflammation within the periurethral prostatic tissue. Nevertheless, specimens for prostatic fibrosis evaluation were acquired from prostate cancer patients rather than from BPH patients, and furthermore, the relationship between each component of MetS and periurethral fibrosis was not elucidated. To the best of our knowledge, our study is the first to investigate the correlation between prostatic fibrosis and MetS with periurethral prostatic tissue harvested from BPH patients. This study has several advantages. First, the data of the included patients were collected prospectively. Second, compared with that in Cantiello and colleagues’ investigation [13], the study cohort was relatively large, as 108 patients were included. Third, we initiated a standardized procedure for prostate tissue procurement, which was carried out at the 3 o’clock position on the left lobe of the prostate prior to the surgery, approximately in the middle of the bladder neck and the verumontanum. We believe that following a standardized procedure could reduce the sampling bias to the maximum extent. Finally, when the correlation between prostatic fibrosis and MetS was revealed, we further studied the correlation between each MetS component and prostatic fibrosis for the first time.

There are some limitations of the current study. First, the inclusion criteria for patients in the study were highly selective, and only BPH patients who were candidates for surgery could be enrolled. The cohort was poorly representative, and we could not study the role of prostatic fibrosis in LUTS or Q_max_. Second, all of the included patients were of Chinese ethnicity and were from two large centers, which may not be fully representative of the entire population and may have introduced a selection bias. Studies have shown significant heterogeneity across ethnic groups, and the results should be interpreted carefully. Third, heterogeneity in the patients’ medication history was not evaluated. In particular, 5α-reductase inhibitors have been reported to change the hormonal status accompanied by tissue fibrosis in the penis as well as in the prostate [27]. Fourth, We did not analyze sex hormone levels because measurement of sex hormones is not typically required in the management of BPH, but it will guide us to improve our clinical work.

## Conclusions

The results of our study suggest that prostatic fibrosis is positively correlated with MetS and its components, including central obesity, elevated fasting glucose, reduced HDL cholesterol and elevated triglycerides, but not with elevated blood pressure. Additional well-designed studies are required to determine the generalizability of these findings to the general population and other ethnicities, as well as to understand the plausible underlying mechanism of the effect of MetS on prostatic fibrosis.

## Data Availability

No datasets were generated or analysed during the current study.
